# Expression of an extracellular ribonuclease gene increases resistance to *Cucumber mosaic virus* in tobacco

**DOI:** 10.1186/s12870-016-0928-8

**Published:** 2016-11-16

**Authors:** Teppei Sugawara, Ekaterina A. Trifonova, Alex V. Kochetov, Yoshinori Kanayama

**Affiliations:** 1 Graduate School of Agricultural Science, Tohoku University, Sendai, 981-8555 Japan; 2 Institute of Cytology and Genetics, SB RAS, Novosibirsk, 630090 Russia; 3 Novosibirsk State University, Novosibirsk, 630090 Russia

**Keywords:** RNA viruses, Plants, Resistance, RNases, Apoplast

## Abstract

**Background:**

The apoplast plays an important role in plant defense against pathogens. Some extracellular PR-4 proteins possess ribonuclease activity and may directly inhibit the growth of pathogenic fungi. It is likely that extracellular RNases can also protect plants against some viruses with RNA genomes. However, many plant RNases are multifunctional and the direct link between their ribonucleolytic activity and antiviral defense still needs to be clarified. In this study, we evaluated the resistance of *Nicotiana tabacum* plants expressing a non-plant single-strand-specific extracellular RNase against *Cucumber mosaic virus*.

**Results:**

Severe mosaic symptoms and shrinkage were observed in the control non-transgenic plants 10 days after inoculation with *Cucumber mosaic virus* (CMV), whereas such disease symptoms were suppressed in the transgenic plants expressing the RNase gene. In a Western blot analysis, viral proliferation was observed in the uninoculated upper leaves of control plants, whereas virus levels were very low in those of transgenic plants. These results suggest that resistance against CMV was increased by the expression of the heterologous RNase gene.

**Conclusion:**

We have previously shown that tobacco plants expressing heterologous RNases are characterized by high resistance to Tobacco mosaic virus. In this study, we demonstrated that elevated levels of extracellular RNase activity resulted in increased resistance to a virus with a different genome organization and life cycle. Thus, we conclude that the pathogen-induced expression of plant apoplastic RNases may increase non-specific resistance against viruses with RNA genomes.

## Background

The apoplast plays an important role in plant defense against various pathogens [1–3]. Ribonucleases belong to the apoplast dynamic proteome and their synthesis increases in response to various stimuli. In particular, some extracellular S-like RNases and pathogenesis-related protein 4 (PR-4) possessing ribonuclease activity are elevated both locally and systemically after wounding or pathogen invasion [4]. It has been assumed that extracellular ribonucleases participate in defense against viruses with RNA genomes. However, a direct link between apoplastic RNA-hydrolyzing activity and resistance against RNA viruses has not been proved. Both PR-4 proteins and S-like RNases have multiple functions, and increased synthesis in response to pathogen invasion may be explained by processes other than direct viral RNA degradation. S-like RNases are considered phosphate-remobilizing enzymes; they digest RNA molecules released from plant cells during senescence or after wounding [5] and some serve as antifungal proteins [6, 7]. Different PR-4 proteins may have several functions (e.g., chitinases, RNases, DNases, and induction of programmed cell death) and are primarily considered as antifungal proteins [8–10]. The expression patterns of both PR-4 and S-like RNase genes are rather complex and their participation in antiviral defense mechanisms is not yet clear.

Non-plant extracellular RNases can be used as a tool to test the direct link between ribonucleolytic activity in the apoplast and resistance against RNA viruses. In this study, we analyzed the resistance of transgenic tobacco plants expressing bovine pancreatic ribonuclease (RNase A) against *Cucumber mosaic virus* (CMV). An elevated level of the heterologous extracellular RNase considerably increased the resistance level. Based on these results and other indirect evidence, we conclude that apoplastic RNases participate in non-specific antiviral defense as part of plant non-host resistance mechanisms [11].

## Methods

Tobacco (*Nicotiana tabacum* cv. SR1) was transformed by a genetic construct containing bovine pancreatic RNase cDNA under the control of the mannopin synthase 2′ promoter [12]. Tobacco plants of three independent transgenic lines (ESR-3, ESR-8, and ESR-10), and wild-type (control) plants were grown using a mixture of vermiculite and perlite (1:1) in plastic pots at 25 °C under artificial light (16 h of light/8 h of dark). A yellow strain of *Cucumber mosaic virus* was used to evaluate virus resistance [13]. Leaves of 4-week-old *Nicotiana benthamiana* plants were rub-inoculated using carborundum with CMV as previously described (Takahashi et al. [14]). After 1 week, the inoculum was prepared by homogenizing the inoculated leaves in 1:5 (w:v) 0.1 M Na-phosphate buffer (pH 7.2) on ice and the homogenate was centrifuged for 10 min at 4 °C. ESR plants and control plants were rub-inoculated with 10 μl of the supernatant, washed with sterile deionized water, and covered with plastic wrap for 1 day. After 10 days, disease symptoms were observed and virus levels in uninoculated upper leaves were analyzed by Western blot using an anti-coat protein polyclonal antibody [14]. RNase activity was measured in the crude extract and the apoplastic fraction according to methods described previously [15, 16].

## Results

### Transgene construction and characteristics of transgenic plants

A genetic construct [12] containing cDNA of the bovine pancreatic RNase gene [17] was cloned in the PC27 vector under the control of the mannopin synthase 2′ promoter [18]. The start-codon context was optimized [19, 20]. The selection of an appropriate promoter was an important step in the design of the genetic construct [21]. The mannopin synthase 2′ promoter is active in roots and leaves, and its activity is strongly induced locally by wounding [22], making it an appropriate choice to mimic S-like RNase gene expression patterns. Previous studies have shown that the bovine RNase pre-protein exhibits correct maturation in plant cells and strongly increases total extracellular ribonuclease activity [12]. Ten kanamycin-resistant independent primary transformants were selected and screened, demonstrating significantly higher levels of ribonuclease activity in crude extracts. Three transformants with stable RNase levels were selected for further analysis (homozygous lines ESR-3, ESR-8, and ESR-10 were verified by a segregation analysis in the T2 generation). The expression of the transgene had no visible effects on plant growth and development.

### CMV accumulation and the development of disease symptoms

**Fig. 1:**
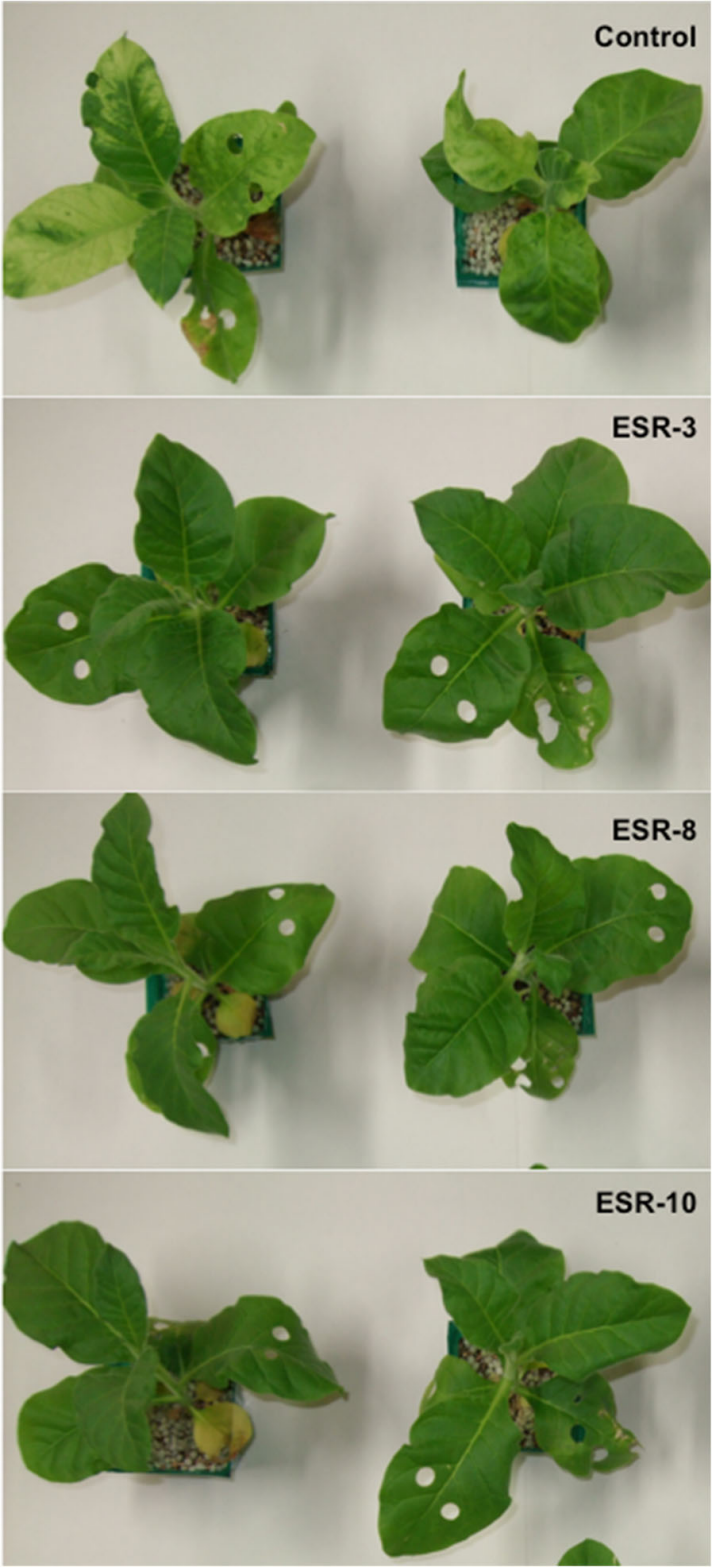
Virus resistance in transgenic (ESR) and control plants. Two typical plants are shown for each ESR line and the control. Disease symptoms were observed in control plants, whereas ESR plants had normal phenotypes

Severe mosaic symptoms and shrinkage were observed in control plants (control) 10 days after inoculation, whereas these disease symptoms were suppressed in ESR transgenic plants expressing the RNase A gene (Fig. 1).

**Fig. 2:**
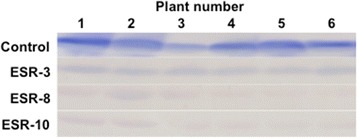
Western blot analysis of the accumulation of CMV after inoculation in ESR and control plants. Protein samples from uninoculated upper leaves were subjected to electrophoresis and blotting. Immunological detection using an anti-CMV coat protein antibody was examined using protein samples from 6 plants in each line

In the Western blot analysis, viral proliferation was observed in the uninoculated upper leaves of control plants, but virus levels were very low in those of the ESR plants (Fig. 2).

The suppression of CMV multiplication was stably observed in each transformant of the three ESR lines. These results suggested that CMV resistance is increased by the expression of the RNase A gene.

**Fig. 3:**
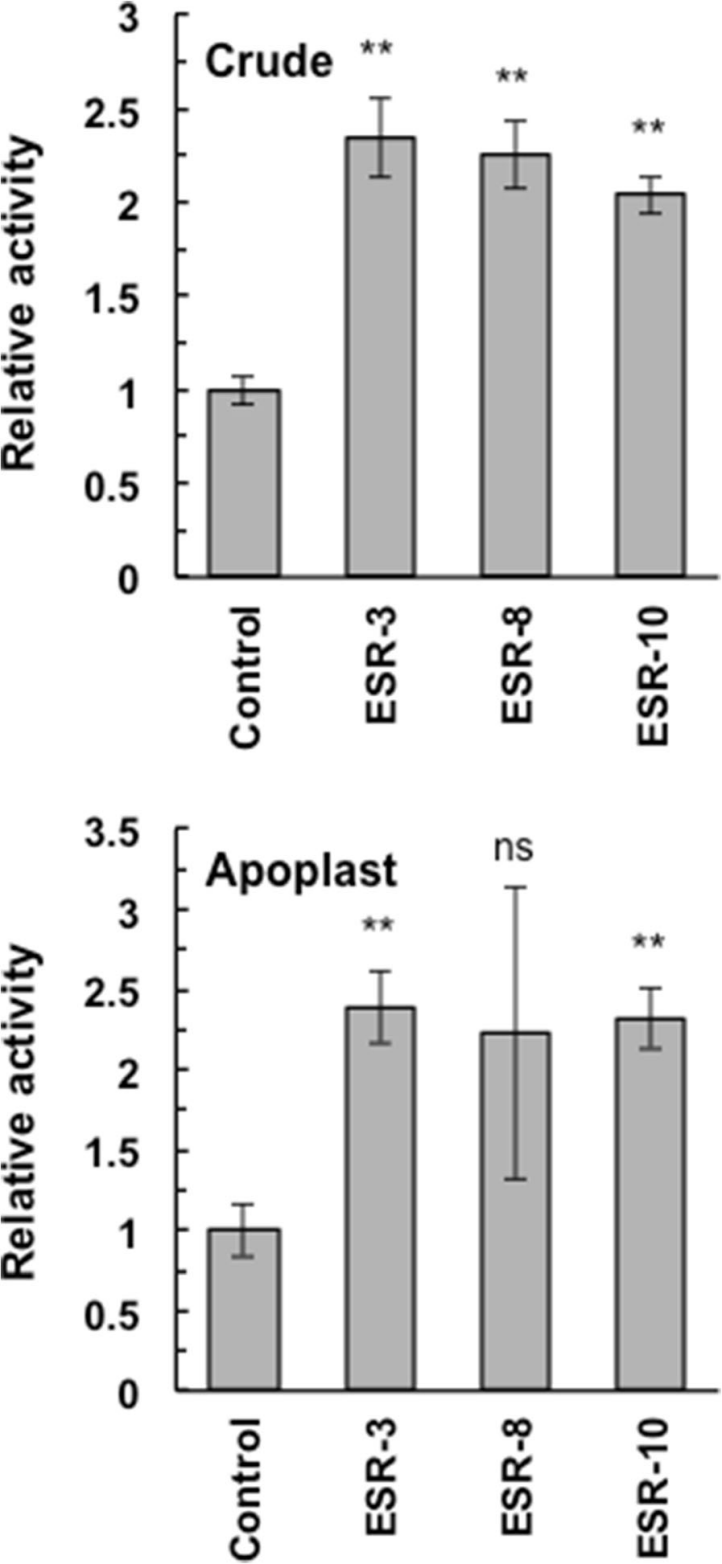
RNase activity in the crude extract and apoplast fractions from ESR and control plants. Relative activity is shown with standard errors (*n* = 8 for the crude extract and *n* = 3 for apoplasts). Significant differences at *P* < 0.01 and no significant differences between ESR and control plants, calculated using the *t*-test, are indicated by ** and ns, respectively

RNase activity was higher in the crude extract and apoplastic fraction obtained from ESR plants than in those obtained from control plants (Fig. 3), further supporting the extracellular localization of the enzyme.

## Discussion

### Plant extracellular proteins with RNase activity

In this study, transgenic plants expressing heterologous RNase in the apoplast were used as a model to investigate the potential role of plant extracellular RNases in the defense against viruses with RNA genomes. Below is a brief description of plant extracellular proteins with RNase activity.

Proteins of the PR-4 family (13–16 kDa) have a conserved C-terminal domain BARWIN, and some have a conserved N-terminal chitin-binding domain (class I PR-4 proteins). Most proteins have an N-terminal signal peptide, and some members of this family also contain a C-terminal signal guiding them into the vacuole [23]. *PR-4* genes usually form small gene families; for example, in rice, five genes arranged in a tandem repeat and characterized by different expression patterns have been found [24].

The expression of different genes in the PR-4 family is induced by very different stimuli, including pathogen invasion, elicitors, tissue wounding, methyl jasmonate, abscisic acid, ethylene, ozone, drought, salinity, cold, UV-light, and heat shock (for a review, see [4]). Some (but not all) PR-4 proteins exhibited both RNase activity and fungicidal properties in the in vitro experiments. Interestingly, mutant protein variants with no RNase activity also lack fungicidal properties (for example, wheat PR4-4 [25], *Ficus pumila* FaPR-4 [26], and *Theobroma cacao* PR-4b [27]). *Malus domestica* PR-4 exhibits both ribonuclease activity specific for single-stranded RNA and significant inhibitory effects against hyphal growth of three apple pathogenic fungi, *Botryosphaeria dothidea*, *Valsa ceratosperma*, and *Glomerella cingulata* [28].

RNase activities of PR-4 proteins are important for their fungicidal functions, but the underlying mechanisms remain unclear. The RNase activity could contribute to fungicidal effects either directly (via the hydrolysis of the mRNA pool of fungi after the penetration of nuclease molecules into the cells of the pathogen), or via the induction of programmed cell death (PCD) at the site of pathogen invasion (hypersensitive response). The mechanism by which RNase molecules penetrate the cells of pathogenic fungi is unknown. It was suggested that the N-terminal hevein-like domain binds chitin and interacts with the lectin of pathogenic fungi to facilitate penetration into fungal cells, whereas the C-terminal RNase domain may be responsible for cytotoxic effects [9]. RNase activity of PR-4 proteins may also be involved in the induction of PCD. For instance, the pepper PR-4c protein is a plasma membrane-localized polypeptide with ribonuclease and proteinase-inhibitor activity that is required for plant cell death and defense signaling [10].

S-like RNases are structurally related to proteins in the T2 family [29]. Some of these enzymes are localized in the apoplast (e.g., RNases RNS1 of *Arabidopsis thaliana*, LE of *Lycopersicon esculentum*, NE of *Nicotiana alata*, Nk of *Nicotiana tabacum*, and ZRNase II of *Zinnia elegans*). It is widely considered that S-like RNases are involved in phosphate remobilization under plant organ senescence or wounding as well as in defense mechanisms against pathogens. Expression of the extracellular RNases RNS1, LE, Nk, and ZRNase II is wound-inducible [30–33]. *A. thaliana* RNS1 is induced upon pathogen invasion both locally (at the site of tissue wounding) and systemically [30]. Tobacco S-like RNase Nk is induced in response to *Cucumber mosaic virus* inoculation [33].

Some S-like apoplastic RNases also exhibit fungicidal activities. The tobacco extracellular RNase NE suppresses the growth of *Phytophthora parasitica* in vitro as well as when administered to the apoplast at the inoculation site. The enzymatically inactive form of the recombinant protein has no fungicidal properties [7]. The molecular mechanisms of fungicidal activity of extracellular S-like RNases are not currently known. It has been hypothesized that RNase molecules can penetrate the cell wall of fungi and destroy cellular RNAs [7].

### Are extracellular RNases involved in antiviral responses?

Extracellular S-like RNases and PR-4 proteins have not been systemically tested as antiviral proteins and their induction after virus infection or wounding can reflect their multiple functions or the complex regulation of plant stress responses [34]. It has been reported that the hypersensitive reaction and local cell death are associated with the synthesis of PR-4 with RNase and DNase activity in *Capsicum chinense* plants that are resistant to Tobamovirus [8]. However, the direct connection of RNA-hydrolyzing activity and antiviral protection still needs to be clarified. We generated transgenic *Nicotiana tabacum* plants expressing heterologous bovine pancreatic RNase (RNase A). These plants were characterized by several-fold higher apoplastic ribonuclease activity than that of control plants (Fig. 3) and RNase A is likely to have no specific functions in plants. Higher RNase levels resulted in increased resistance to CMV, indicating a role of RNA-hydrolyzing enzymes in the plant antiviral response. Indeed, RNase A may occasionally have specific activity against CMV or CMV genomic RNA molecules can be specifically exposed and easily digestible. However, our previous results demonstrated increased resistance of RNase A-expressing tobacco plants against tobacco mosaic virus (TMV) [12].

CMV and TMV belong to distinct families and have different genome organizations and life-strategies. TMV (Virgaviridae, Tobamovirus) has a rod-like capsid and enters cells by mechanical wounding, which either opens the plasma membrane or allows pinocytosis [35]. The virion rapidly disassembles and the cellular protein synthesis machinery initiates translation of positive-sense TMV genomic RNA encoding four viral proteins. Newly synthesized virus particles move from cell-to-cell through plasmodesmata and initiate new cycles of replication. Finally, they reach the vascular system for rapid systemic spread through the phloem to other parts of the infected plant (for details, see [35]). The CMV (Bromoviridae, Cucumovirus) genome consists of three different positive-sense RNAs packed into separate spherical particles. It can be transmitted from plant to plant both mechanically by sap and by aphids in a stylet-borne (non-persistent) fashion. Cell-to-cell CMV movement also occurs through plasmodesmata and long-distance movement occurs through the phloem (for details, see [36] or elsewhere).

There are several possible mechanisms of apoplastic RNase-mediated antiviral effects. They can digest viral genomic RNA if it is exposed on the virion surface. They may also penetrate the plant cell from the apoplast together with viruses and hydrolyze viral genomic RNA molecules immediately after their release from the virion. Finally, viruses frequently penetrate plant cells through wounded surfaces. If apoplastic RNases penetrate the cytoplasm through damaged cell walls, they can kill the cell via mRNA and/or rRNA digestion, thereby decreasing the number of cells susceptible to virus replication. The inoculation of RNase-expressing tobacco transgenic plants in experiments was performed by mechanical wounding ([12]; this manuscript), which mimics the transmission mechanisms of TMV and CMV in nature. Thus, it is likely that extracellular S-like RNases and PR-4 proteins also participate in plant non-specific defense mechanisms against viruses with RNA genomes. Interestingly, buckwheat plant cultivars characterized by relatively high ribonuclease activity in leaf extracts are also relatively highly resistant to buckwheat burn virus (*Rhabdoviridae*) [37].

## Conclusion and perspectives

It is known that plant extracellular RNases play important roles in various physiological processes, including resistance to pathogenic fungi. Here, we demonstrated that increased RNase activity in the apoplast is correlated with high non-specific resistance to viruses with RNA genomes. Accordingly, the functions of apoplastic RNases need to be extended to include plant antiviral defense as a part of the basal or non-host resistance mechanisms [11].

Resistance to some viruses has also been evaluated in transgenic plants expressing a double-strand-specific RNase gene [38]. Enhanced resistance to tomato mosaic virus, CMV, potato virus Y, and tomato spotted wilt virus has been reported in tobacco, impatiens, and chrysanthemum plants expressing the *pac1* ribonuclease gene from *Schizosaccharomyces pombe* [39–41]. Although the expression of a double-strand-specific RNase gene can induce resistance to some viruses, various levels of resistance have been observed, from only a delay in the appearance of disease symptoms to nearly complete protection. Resistance to some viruses has been evaluated in tobacco plants expressing a bacterial double-strand-specific RNase gene [42], and the transgenic plants were resistant to infection by viruses with a divided RNA genome, but not by viruses with a single RNA genome.

These observations emphasize the application of genes encoding extracellular RNases as suitable tools for crop improvement. For example, genetic markers associated with increased levels of apoplast ribonuclease activity can be used for marker-oriented breeding targeting high resistance to viruses and fungi [37, 43]. Transgenes encoding either plant or heterologous extracellular RNases can be used for prospective GM-crop production ([12, 44], this paper]. It seems likely that an apoplastic “ribonuclease barrier” provides an aggressive environment for viruses with respect to penetration and strengthens non-specific resistance mechanisms.

The results of induced resistance to CMV and TMV are meaningful per se because these viruses are among the top five most important viruses from academic and industrial perspectives [45]. Therefore, transformation using an extracellular single-strand-specific RNase gene could be a promising strategy for the engineering of plants with resistance to a wide range of viruses. This technology should be established by additional evaluations of resistance to other viruses and viroids, to which resistance has been reported in transformation studies using double-strand-specific RNase genes [41, 46].
